# Multiple Components of the VHL Tumor Suppressor Complex Are Frequently Affected by DNA Copy Number Loss in Pheochromocytoma

**DOI:** 10.1155/2014/546347

**Published:** 2014-09-14

**Authors:** David A. Rowbotham, Katey S. S. Enfield, Victor D. Martinez, Kelsie L. Thu, Emily A. Vucic, Greg L. Stewart, Kevin L. Bennewith, Wan L. Lam

**Affiliations:** ^1^Department of Integrative Oncology, BC Cancer Agency, Vancouver, BC, Canada; ^2^BC Cancer Research Centre, 675 West 10th Avenue, Vancouver, BC, Canada V5Z 1L3

## Abstract

Pheochromocytomas (PCC) are rare tumors that arise in chromaffin tissue of the adrenal gland. PCC are frequently inherited through predisposing mutations in genes such as the von Hippel-Lindau (*VHL*) tumor suppressor. VHL is part of the VHL elongin BC protein complex that also includes CUL2/5, TCEB1, TCEB2, and RBX1; in normoxic conditions this complex targets hypoxia-inducible factor 1 alpha (HIF1A) for degradation, thus preventing a hypoxic response. *VHL* inactivation by genetic mechanisms, such as mutation and loss of heterozygosity, inhibits HIF1A degradation, even in the presence of oxygen, and induces a pseudohypoxic response. However, the described <10% VHL mutation rate cannot account for the high frequency of hypoxic response observed. Indeed, little is known about genetic mechanisms disrupting other complex component genes. Here, we show that, in a panel of 171 PCC tumors, 59.6% harbored gene copy number loss (CNL) of at least one complex component. CNL significantly reduced gene expression and was associated with enrichment of gene targets controlled by HIF1. Interestingly, we show that VHL-related renal clear cell carcinoma harbored disruption of *VHL* alone. Our results indicate that VHL elongin BC protein complex components other than VHL could be important for PCC tumorigenesis and merit further investigation.

## 1. Introduction

Von Hippel-Lindau (VHL) disease is a rare inherited syndrome which predisposes individuals to a variety of malignant and benign tumors including renal cell carcinoma and pheochromocytoma (PCC) [1]. Renal cell carcinomas are cancers of the kidney that account for approximately 102,000 deaths worldwide each year [2, 3]. Renal clear cell carcinoma (RCC), arising in the proximal convoluted tubules of the kidney transport system, is the most common subtype of renal cell carcinomas (comprising about 88% of tumors) and is tightly associated with inactivating mutations of the* VHL* gene [4, 5]. PCC, the other principal VHL-related cancer, is a rare catecholamine-secreting cancer originating in chromaffin cells of the adrenal gland [6–8]. Although these tumors can be benign, the malignancy rate ranges from 10 to 15%. Malignant PCC is identified histologically by the presence of metastasis (commonly to lymph nodes, liver, lungs, and bone). Patients with malignant PCC have a high risk of mortality and morbidity. The overall 5-year survival rate of malignant PCC is 40–77% [9–11]. Therefore, a greater understanding of the biology underlying PCC is needed in order to advance diagnostic testing and prognosis.

In approximately one-third of cases, PCC arises in patients with germ-line mutations in predisposing genes such as* VHL*,* NF-1*,* MEN2/RET*, and* SDH* subunits,* TMEM127*,* MAX*, or* HIF2A*, among others [12–14]. Studies indicate that* VHL* is among the most frequently targeted genes in PCC, mostly affected by genetic mechanisms such as mutations and loss of heterozygosity (LOH) [15–17]. In keeping with Knudson's two-hit hypothesis [18], tumors from patients who have a germ-line mutation in one* VHL* allele are susceptible to somatic inactivation of the remaining allele. Indeed, studies show that a somatic “second hit,” which can arise through epigenetic or genetic mechanisms, results in a loss of* VHL* gene expression, abnormal VHL protein function, and consequent tumorigenesis [19–21].

**Figure 1:**
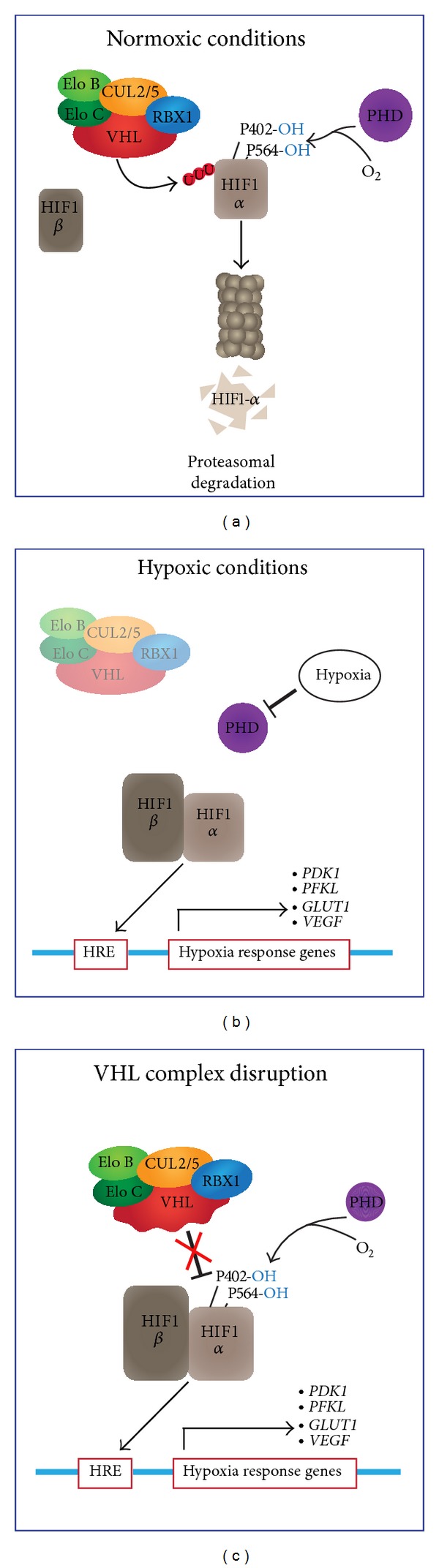
Schematic illustration of the role of the VHL elongin BC complex in the HIF1 pathway in normoxic, hypoxic, and pseudohypoxic conditions. Under normal physiological conditions (a), HIF1-*α* becomes hydroxylated on two prolyl residues. Hydroxylation of HIF1-*α* generates a binding site for the VHL elongin BC complex, consisting of elongin B, elongin C, CUL2 or CUL5, RBX1, and VHL, which directs the polyubiquitination of HIF1-*α* and targets it for proteasomal degradation [35]. In hypoxic conditions (b), PHD proteins no longer hydroxylate HIF1-*α* and VHL cannot add destabilizing ubiquitin polymers to HIF1-*α*. HIF1-*α* can then heterodimerize with HIF1-*β* and translocates into the nucleus where it binds to hypoxia response elements (HRE) and promotes the expression of genes, such as* PDK1*,* PFKL*,* GLUT1*, and* VEGF*, that mediate the cellular response to hypoxic conditions. Similarly, in some cancer types, such as PCC and RCC (c), a loss of function event (such as DNA sequence mutation or copy number loss) of VHL can result in an upregulation of HIF1-target genes independent of the oxygenation status of the tumor cells.

VHL is a component of the VHL elongin BC complex—composed of the proteins VHL, CUL2 or CUL5, RBX1, and elongin B/elongin C (elongins B and C are encoded by* TCEB1* and* TCEB2,* resp.). This complex acts as an E3 ubiquitin-ligase and drives the proteasomal degradation of targeted proteins [22, 23]. The hypoxia-inducible factor 1*α* (HIF1-*α*, encoded by* HIF1A*), the primary target of this complex, regulates over 80 genes associated with tumor progression, glycolysis, angiogenesis, and metastasis and is negatively regulated by the VHL elongin BC complex [24, 25]. Hypoxia inducing factor 1 (HIF1) is composed of an alpha subunit, which is negatively regulated by the VHL elongin BC complex and a beta subunit, which is constitutively expressed [26]. Under normoxic conditions, the hydroxylation of HIF1-*α* at two prolyl residues (P402 and P564) by PHD-containing proteins creates a binding site for* VHL* and results in proteasomal degradation of HIF1-*α* (Figure 1(a)) [27–29]. In hypoxic conditions, PHD-containing proteins no longer hydroxylate HIF1-*α* and VHL cannot add destabilizing ubiquitin polymers to HIF1-*α*. HIF1-*α* can then heterodimerize with HIF1-*β* and translocate into the nucleus where it binds to hypoxia response elements (HRE) and promotes the expression of genes, such as* PDK1*,* PFKL*,* GLUT1*, and* VEGF*, that mediate the cellular hypoxic response. Genetic alterations affecting* VHL* or other complex components can lead to abnormal stabilization of HIF1-*α*, resulting in aberrant translocation of HIF1 to the nucleus and ectopic activation of target genes, such as* VEGF*,* PDK*, and* EPO*, to elicit a hypoxic response, even in normoxic conditions (Figure 1(c)) [30–32].

Previous studies of PCC have reported that disruption of the VHL elongin BC protein complex occurs through gene copy number loss, mutation, or epigenetic silencing of the* VHL* gene and that this disruption leads to tumorigenesis through activation of HIF1 targets [16, 20, 33, 34]. The role of the other components of the VHL elongin BC complex is largely uncharacterized. In the present study, we investigated DNA-level alterations—gene copy number losses (CNL) and promoter hypermethylation—affecting other components of the VHL elongin BC protein complex in PCC. We assessed the effects of these alterations at the gene expression level and the impact of complex component disruption on enrichment of HIF1-target expression. Finally, we explored whether similar disruptions were present in another* VHL*-inactivated cancer type, RCC. Our results indicate that, while* VHL* is disrupted in both PCC and RCC, other components of the VHL elongin BC complex, particularly* RBX1* and* CUL5*, are significantly disrupted in PCC and their status might be an important clinical consideration in PCC.

## 2. Materials and Methods

### 2.1. Pheochromocytoma DNA Copy Number Data Analysis

Information regarding DNA copy number alterations affecting VHL tumor suppressor complex components (*VHL*,* TCEB1*,* TCEB2*,* RBX1*,* CUL2*, and* CUL5*) was obtained from 171 PCC tumors available through The Cancer Genome Atlas Project (TCGA). Gene dosage alterations were assessed using the Affymetrix SNP6.0 platform at the Broad TCGA Genome Characterization Center [36]. Processed level 3 data was accessed through the UCSC Cancer Genome Browser [37–39]. Briefly, raw copy number data was segmented using a circular binary segmentation algorithm [40] and mapped to hg18 genome assembly. In order to exclude polymorphic variations, a fixed set of common germ-line copy number variant probes were removed prior to segmentation. Coordinates were converted to hg19 using a local repository of galaxy, running the LiftOver utility [41].

Segmented data was loaded into the Integrative Genomics Viewer (IGV) [42, 43], and information regarding the six complex component genes was exported as a.tdm file. DNA copy number alterations were defined as follows: (1) DNA copy number loss (signal intensity log⁡⁡2 ratio < −0.3), copy number neutral (log⁡⁡2 ratio between −0.3 and 0.3), or copy number gain (log⁡⁡2 ratio > 0.3).

### 2.2. Pheochromocytoma Gene Expression Data Analysis

Gene-level transcription estimates, in the form of RSEM normalized counts, were obtained for the six complex component genes analyzed and were obtained from processed RNA sequencing data derived from 171 tumors and 4 adjacent nonmalignant tissues [44]. Gene expression profiles were generated using the Illumina HiSeq 2000 RNA sequencing platform by the University of North Carolina and TCGA Genome Characterization Center. Individual expression profiles were loaded into IGV, and expression information for each gene was exported. Genes were mapped to the human genome hg19 coordinates using UCSC cgData HUGO probeMap.

### 2.3. Pheochromocytoma DNA Methylation Data Analysis

Methylation analyses using the Illumina Infinium HumanMethylation450 platform were performed at Johns Hopkins University, University of Southern California, and TCGA genome characterization center. Probes mapping to the six complex component genes were extracted. Only probes mapping to the promoter region, which are most likely to have an effect on gene expression, were selected for further analysis. The ratio of the intensity of the methylated bead type to the combined locus intensity (termed as beta values (*β*V)) was calculated using BeadStudio software. To assess the difference in probe methylation between PCC and nonmalignant tissue, a delta beta value (d*β*V) was calculated for each probe: an average *β*V was calculated for each probe in the nonmalignant cohort, and these values were subtracted from the PCC *β*Vs on a tumor-by-tumor basis.

### 2.4. Pheochromocytoma and Renal Clear Cell Carcinoma DNA Mutation Analysis

Somatic mutation data using Illumina sequencing platforms were obtained from TCGA. Data was derived from 171 PCC tumors samples that also contained expression and copy number information.* VHL* complex component genes (*CUL2*,* CUL5*,* TCEB1*,* TCEB2*, and* RBX1*) as well as 3 genes known to be frequently mutated in PCC (*RET, HRAS*, and* NF1*) were classified as having an inactivating mutation if the result was a frame shift insertion, frame shift deletion, splice site mutation, missense mutation, or a nonsense mutation. Somatic mutation data was also analyzed in the same way in a cohort of 417 out of 522 RCC tumors where DNA sequence data was available.

### 2.5. Renal Clear Cell Carcinoma DNA Copy Number Data Analysis

Copy number data and mutation data for 522 RCC were downloaded from cBioPortal for Cancer Genomics (http://www.cbioportal.org) [45, 46]; of these, 411 samples had concurrent copy number and mutation data. The same criteria used for PCC were applied to define copy number loss and gain. Mutations with a neutral or low mutation assessor score were not considered in mutation frequency calculations.

### 2.6. Correlation of DNA-Level Alterations with Gene Expression in Pheochromocytoma

In order to assess the effect of DNA-level alteration on gene expression of the VHL tumor suppressor complex components, PCC was divided into up to three groups based on copy number alteration status (copy number loss, copy number neutral, and copy number gain) and expression was compared between groups using GraphPad software v6. For most genes (*VHL*,* CUL5*,* TCEB1*, and* TCEB2*) three group comparisons were performed using a Kruskal-Wallis test. Since the majority of copy number alterations were CNL rather than gain, a two-group comparison was also performed for each of the six genes comparing CNL and neutral copy number using a Mann-Whitney *U* test.* RBX1* did not show copy number gain in any sample; therefore only a Mann-Whitney *U* test was performed for this gene. In all comparisons, a *P* value < 0.05 was considered significant.

Correlation between gene expression and promoter hypermethylation was assessed through Spearman correlation analysis using GraphPad software v6. Each probe was correlated separately using a gene expression matrix of the 171 PCC samples. An example of the correlation of the probe, cg07288693, located in the promoter region of* RBX1*, is shown in Supplementary Figure 1 Supplementary Material available online at http://dx.doi.org/10.1155/2014/546347.

### 2.7. Gene-Set Enrichment Analysis

In order to assess possible effects of HIF1-target genes due to the disruption of the VHL elongin BC tumor suppressor complex, we evaluated a gene-set enrichment analysis (GSEA) for every sample using the single sample gene-set enrichment analysis (ssGSEA). Briefly, ssGSEA calculates separate enrichment scores (ES) for each pairing of a sample and gene set. Each ssGSEA ES represents the degree to which the genes in a particular gene set are coordinately up- or downregulated within a sample [47]. A rank normalized expression matrix for 171 PCC samples and 20,533 genes was used as input on the ssGSEA implementation in GenePattern public server [48]. ssGSEA was performed using default parameters using the SEMENZA_HIF1_TARGETS gene set available from the Molecular Signatures Database v4.0 (Broad Institute). This gene set contains 36 genes that are transcriptionally regulated by hypoxia-inducible factor 1 (HIF1) [49]. ES for each sample are available in Supplementary Table 2.

## 3. Results

### 3.1. Inactivation of VHL Elongin BC Complex Components in Pheochromocytoma

**Figure 2:**
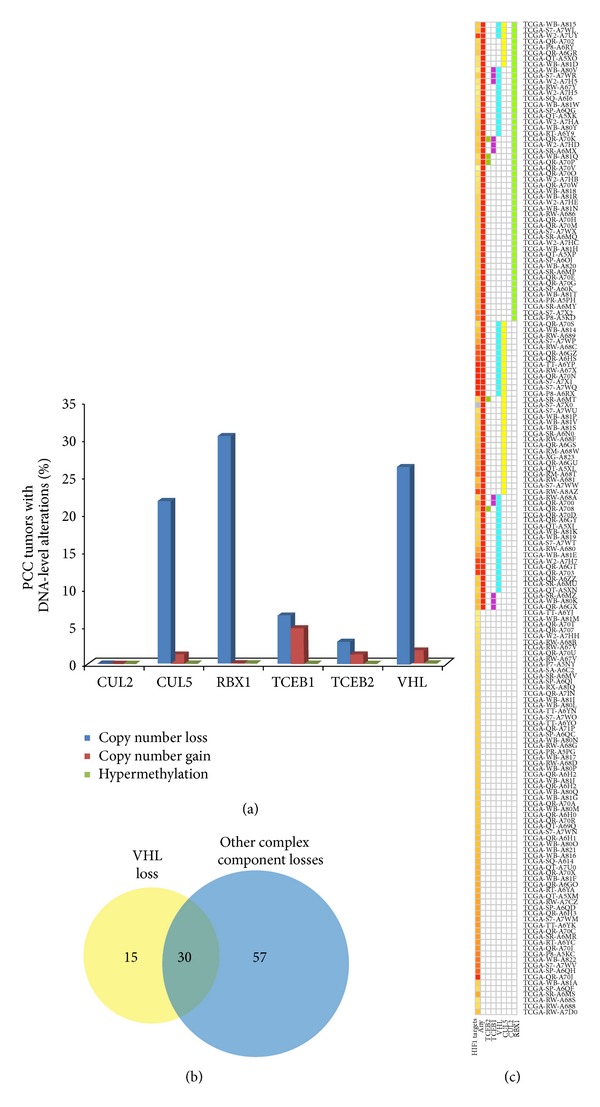
Disruption of VHL complex component genes. (a) Frequency of DNA copy number alterations and promoter hypermethylation for each component of the VHL elongin BC complex. (b) Venn diagram detailing PCC samples that have copy number loss of VHL complex components either alone or in combination. (c) Incidence of copy number loss of individual components of the VHL complex (*RBX1, CUL2, CUL5, TCEB1, TCEB2,* and* VHL*) across a panel of 171 pheochromocytomas. Each row represents an individual complex component affected by copy number loss:* RBX1* (light green),* CUL5* (yellow),* TCEB1* (pink),* TCEB2* (dark green), or* VHL* (aqua). Presence of any alteration in any individual component is shown in red. The bottom row (yellow to red gradient) indicates the gene enrichment score HIF1 gene targets with red bands representing greater enrichment. All samples appear to be enriched for HIF1 targets though not all samples have loss of function events affecting* VHL*.

We first examined the mutation status of the* VHL* gene in 241PCC tumors from the Catalogue of Somatic Mutations in Cancer (COSMIC). Consistent with literature reports, 24 out of 241 (10%) cases harbored* VHL* gene mutation [17]. The data from TCGA also showed a very low frequency of* VHL* mutation, at 2%. We next analyzed the copy number status of component genes:* VHL*,* RBX1*,* CUL2*/*CUL5*,* TCEB1*, and* TCEB2*. In a cohort of 171 PCC tumors from The Cancer Genome Atlas (TCGA), the frequency of gene disruption for three of these complex components was remarkably high (*RBX1*, 30.4%;* VHL*, 26.9%;* CUL5*, 21.6%), while two remaining complex components exhibited the modest disruption frequencies in PCC tumors:* TCEB1* (6.4%) and* TCEB2* (2.9%).* CUL2* did not exhibit any gene CNL according to our parameters. Interestingly, copy number gains were infrequent in all complex component genes; no gene displayed gain in more than 5% of cases (Figure 2(a)).

Strikingly, when complex gene disruption was considered cumulatively, 59.6% of PCC harbored genetic loss of at least one of the complex components, while 24.3% harbored disruption of 2 or more complex components (Figure 2(c)). Gene CNL events were not mutually exclusive: 17.5% of PCC had genetic loss of both* VHL* and any other complex component; 33.3% harbored loss of a complex component other than* VHL*, and 8.8% harbored loss of* VHL* alone (Figure 2(b)). These findings highlight the importance of VHL elongin BC protein complex disruption at the genetic level in PCC.

We next investigated whether gene silencing by aberrant DNA methylation affected any of the complex component genes. Gene methylation data was available for 171 PCC tumors and 4 adjacent solid nonmalignant tissues. We found that all but one probe (cg03160045) had a *β*V of less than 0.14 in both PCC and nonmalignant samples, indicating that complex component genes were not highly methylated. Further, the d*β*V (“PCC *β*V” and “nonmalignant *β*V”) were close to zero in all cases, for all probes, suggesting that methylation of complex component genes did not differ between tumor and nonmalignant samples. Interestingly, we found that methylation was significantly negatively correlated with* RBX1* expression (Supplementary Figure 1); however, given the low d*β*V for all probes, we could not confidently attribute the effects of methylation to expression levels of* RBX1*. Therefore, for the remainder of the analysis, we focused on gene CNL.

### 3.2. Enrichment of HIF1-*α* Target Genes in Pheochromocytoma

We next examined whether genetic disruption of other complex component genes might lead to overactivity of HIF1 and aberrant expression of HIF1-target genes. We performed single sample gene-set enrichment analysis (ssGSEA) on the panel of 171 PCC and found that positive HIF1-target gene-set enrichment (Supplementary Table 1) occurred to some degree in all cases (median ES = 6132.4) (Figure 2(c), Supplementary Table 2). Gene CNL of at least one complex component could explain HIF1-target gene enrichment in 59.6% of cases. Since 33.1% of cases harbored genetic loss of VHL components other than* VHL*, and TGCA mutation data indicated a* VHL* mutation rate of only 2%, inactivation of other VHL elongin BC complex components is likely involved in VHL elongin BC complex dysfunction in PCC and this could impact HIF1-target expression (Figures 2(b) and 2(c)).

### 3.3. Gene Dosage Affects Expression of VHL Elongin BC Complex Components in PCC

**Figure 3:**
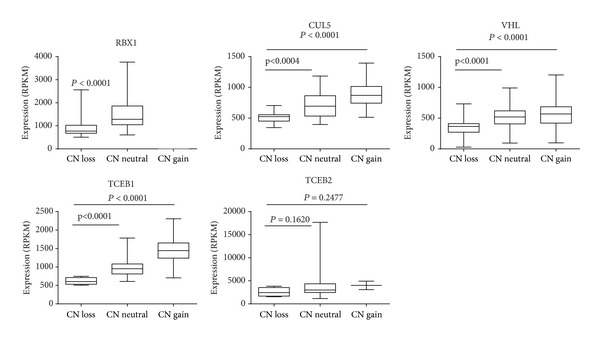
Gene dosage of VHL elongin BC complex components affects expression in PCC.* RBX1* expression is positively correlated with copy number loss (Mann-Whitney *P* value < 0.0001) and no copy number gain was seen for* RBX1* in PCC samples. Copy number was significantly positively correlated with expression for* CUL5*,* VHL*, and* TCEB1* complex components (Kruskal-Wallis *P* value < 0.0001).

We next evaluated whether gene copy number alterations to the VHL elongin BC complex components were correlated with expression of these genes. RNA sequencing data for the 171 PCC tumors was downloaded from TCGA, and samples were grouped according to their copy number status. Intriguingly, gene dosage in four of the five VHL complex components that were altered at the copy number level—*RBX1*,* CUL5*,* VHL*, and* TCEB1*—was significantly positively correlated with expression (*P* < 0.0001) (Figure 3).* TCEB2*, which had a gene CNL frequency of 2.9%, was not significantly correlated with expression;* CUL2* was not altered at the copy number level. These findings suggest that underexpression of* RBX1*,* CUL5*,* VHL*, and* TCEB1* is a selected event in PCC.

### 3.4. DNA-Level Alterations Affecting VHL Elongin BC Complex Components Differ between VHL-Related Cancers

**Figure 4:**
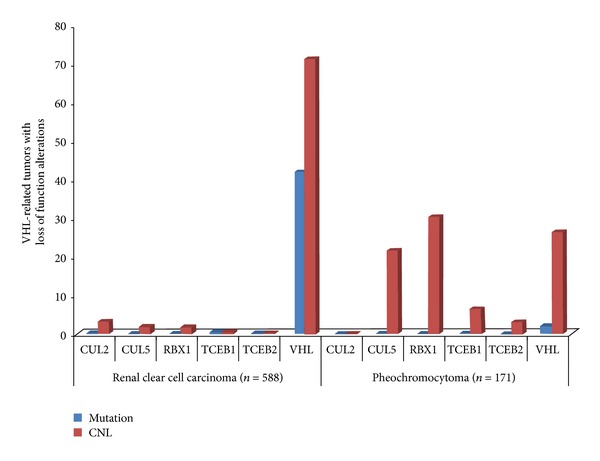
Frequency plot displaying two VHL-related tumors, renal clear cell carcinoma and pheochromocytoma, and corresponding types of DNA-level alterations that affect each individual complex component. The figure shows patterns and frequency of DNA copy number loss (red) and mutation (blue) affecting each component of the VHL complex in the two tumor types.

We evaluated if gene CNL affects components of the VHL elongin BC protein complex in another cancer type characterized by inactivation of* VHL*: RCC. We queried gene CNL and mutation frequencies of the six VHL elongin BC complex genes in RCC using resources available at the cBioPortal for Cancer Genomics. In RCC, negligible gene CNL and mutation frequencies were observed for all of the VHL complex genes except for* VHL* itself (Figure 4). Across 522 RCC tumors,* VHL* was mutated in 179 (34.3%) samples and lost at the copy number level in 419 (71.3%) samples, while 39.7% of samples exhibited concurrent mutation and CNL,* RBX1*,* CUL2*,* CUL5*,* TCEB2*, and* TCEB2* did not appear to be significantly altered in RCC at DNA level, with no complex components exceeding a CNL frequency of 4% and no mutation frequency reaching 1%.

By contrast,* VHL* mutation frequency in PCC was only 2% according to TCGA data and, of the 171 PCC tumors from TCGA, only 26.3% of samples exhibited CNL of* VHL*, with* RBX1* and* CUL5* also undergoing frequent CNL. From these data, we observe markedly different genetic profiles when comparing genes coding for the VHL elongin BC complex between RCC and PCC. In RCC, it appears that* VHL* is the sole contributor to disruption of VHL elongin BC complex, whereas, in PCC, VHL elongin BC complex loss of function may occur frequently through CNL of* VHL*,* RBX1*, and* CUL5* and through mutation of* VHL*.

### 3.5. Correlation of VHL Elongin BC Protein Complex Status with Other Frequent Somatic Mutations in PCC

We evaluated the mutation status of three other genes known to be frequently mutated in PCC (*HRAS*,* RET,* and* NF1*) to assess their relationship with alterations in* VHL* complex component genes [13, 50]. Of the 171 PCC tumors analyzed, 39 had mutations in either* HRAS*,* RET,* or* NF1* (Supplementary Figure 2). Twelve of the 39 cases had no disruption in the* VHL* complex, while 27 displayed VHL elongin BC protein complex disruption. The frequency of cases harboring mutations in* RET*,* HRAS,* or* NF1* did not significantly differ between cases with or without VHL complex disruption (*P* = 0.1942, chi-square test). In RCC,* NF1*,* RET,* and* HRAS* were not frequently mutated (2%, 0%, and <1%, resp.), and these mutations were not mutually exclusive with* VHL* mutations.

## 4. Discussion

Oxygen-sensing pathways are paramount for cell survival and normal cellular function, while they also play a key role in tumor progression and aggressiveness. HIF1 pathways allow cells to survive in conditions of temporary oxygen deprivation (e.g., HIF is essential in embryonic development). Since abnormal accumulation of HIF1-*α* subunits can induce HIF1 pathways to promote tumor progression and aggressiveness, its levels need to be tightly regulated. This function is mainly achieved by the VHL elongin BC complex [28]. In VHL-related tumors, such as PCC and RCC [20, 34], HIF1 activity is aberrantly and constitutively high, mimicking a hypoxic environment, irrespective of oxygen levels [31, 51, 52]. Genetic lesions affecting the* VHL* gene are usually considered the cause of HIF1-*α* accumulation [25, 26].

Since genetic mechanisms disrupting the* VHL* gene are only present in a fraction of PCC, we have tested the hypothesis that deregulation of other protein components of the VHL elongin BC complex might also result in activation of HIF1 pathways. Indeed, gene CNL affecting* RBX1* and* CUL5* significantly impacted gene expression. We also noticed a negative correlation between hypermethylation and expression for* RBX1*; however, the low methylation levels of probes across all samples, including nonmalignant samples, imply that hypermethylation is not a major mechanism of* RBX1* downregulation. It has been shown that underexpression of* RBX1*, due to gene* CNL*, might interfere with the* KEAP1*/*CUL3*/*RBX1* complex, which also displays a E3 ubiquitin-ligase activity in thyroid and ovarian cancer [53]. Similarly, underexpression of* CUL5* linked to genetic loss events has been documented in breast tumors [54]. Together, these results suggest that gene CNL has an impact on decreased gene expression of* RBX1* and* CUL5*, in addition to* VHL*, and may subsequently contribute to dysfunction of the VHL elongin BC complex in PCC. Interestingly, other frequent somatic mutations in PCC, such as those affecting* HRAS*,* RET,* and* NF1*, seem to occur independently of VHL elongin BC protein complex disruption.

It has been well documented that loss of function alterations to* VHL*, components of the succinate dehydrogenase (SDH) complex, and HIF2A, as well as pseudohypoxia characterize cluster 1 PCC tumors and correlate with increased HIF1 signaling [25, 26, 55, 56]. We propose the fact that tumors involving DNA-level alterations of the VHL complex component genes should be considered as part of this cluster. An enrichment analysis (GSEA) of the panel of 171 PCC tumors revealed that HIF1 gene targets were positively enriched in all cases (Figure 2(c)). The high frequency of samples showing alterations in at least one of the complex components (59.6%) at least partially explains the positive enrichment of HIF1 targets. In cases that did not have clear DNA-level alterations to members of the VHL elongin BC complex, upregulation of HIF1-target genes may simply be due to the presence of hypoxic cells in the biopsy of samples used to generate the data. From these observations, we suggest that gene CNL of other VHL complex components, namely,* RBX1* and* CUL5*, along with* VHL,* CNL, and mutation, facilitates dysfunction of this complex and the consequent accumulation of HIF1-*α*.

*VHL* is also frequently disrupted in RCC. Previous studies have shown that the* VHL* gene is affected by somatic mutations in 50% of cases, while hypermethylation is observed in 10–20% of sporadic RCC [20]. We analyzed disruption of* VHL* and other complex components in RCC and compared these results to their disruption in PCC. Interestingly,* VHL* seems to be the only gene significantly disrupted in RCC, with 71.3% of cases undergoing CNL and other 34.3% with* VHL* mutation. These results indicate that RCC tumors are likely dependent on elimination of* VHL* rather than other complex components in order to generate conditions of pseudohypoxia. The genetic landscape of the VHL elongin BC complex genes in PCC, however, showed* VHL* gene to be less frequently inactivated at the DNA level, with the burden of genetic inactivation of the VHL elongin BC complex seeming to fall somewhat equally on* RBX1*,* CUL5,* and* VHL*.

The data presented here provide a rationale for a more comprehensive interrogation of the role of other* VHL* complex components (namely,* RBX1* and* CUL5*) in the HIF1-mediated oxygen-sensing pathway in PCC. In summary, we present compelling evidence that HIF1-mediated pseudohypoxic conditions are genetically selected in PCC via the disruption of multiple VHL complex components and we provide further rationale for exploring this pathway as a therapeutic target in PCC with potential application to RCC and other VHL-related diseases.

## Supplementary Material

Supplementary material provides a sample correlation plot for methylation, copy number and expression data in PCC tumors; the incidence of VHL elongin BC protein complex component gene disruption in the context of other mutations common in PCC; the gene enrichment score for the cohort of PCC tumors and a list of HIF1α gene targets. Supplementary figure 1 shows that *RBX1*, a VHL elongin BC complex component, is hypomethylated and that aberrant DNA methylation does not appear to be a major mechanism of deregulation. Supplementary figure 2 highlights that mutations in *NF1*, *HRAS* and *RET* (other genes commonly mutated in PCC) occur independent of VHL elongin BC protein complex status. Supplementary tables detail the level of HIF1 enrichment in each PCC sample and the genes that are transcritionally regulated by HIF1.
